# SOCIAL CONNECTIONS AND HEALTH AMONG OLDER ADULTS LIVING IN SUBSIDIZED HOUSING: LOCAL STAKEHOLDERS PERSPECTIVES

**DOI:** 10.1093/geroni/igac059.2630

**Published:** 2022-12-20

**Authors:** Avery Bullock, Joseph Gallo, Carl Latkin, Cynthia Boyd, Thomas Cudjoe

**Affiliations:** Johns Hopkins University School of Medicine, Baltimore, Maryland, United States; Johns Hopkins University, Baltimore, Maryland, United States; Johns Hopkins University, Baltimore, Maryland, United States; Johns Hopkins University, Baltimore, Maryland, United States; Johns Hopkins School of Medicine, Baltimore, Maryland, United States

## Abstract

Social isolation among older adults living in the United States is a major public health problem that disproportionately affects those living below the poverty line. Though decades of epidemiological studies have provided important insights on the impact of social isolation on health, there have been limited qualitative studies on the dynamics of social connections among older adults in subsidized housing. This study aims to advance our understanding of the perspectives of local stakeholders in the field of subsidized housing on the social connections of older adults and how to enhance social connection. Semi-structured interviews were conducted between July 2021 and October 2021 with local stakeholders in Baltimore’s subsidized housing community to better understand social connections among older adults. Stakeholders included, but were not limited to, the following positions: service coordinators, property managers, maintenance staff, and security guards. Interviews were imported and analyzed using NVIVO12 software and a qualitative content analytic approach. Preliminary themes identified included: barriers and facilitators of connection, engagement with family, and ideas about interventions. Mental and physical health challenges, access to and use of communications technology, and lack of transportation were barriers while food incentives, gift card giveaways, and resident champions were facilitators of social connection. Social isolation among older adults is an important concern among stakeholders in subsidized housing. To promote social connection in older adults, interventions must adopt a multi-pronged approach that addresses the barriers and leverage existing facilitators within subsidized housing communities.

